# Supplementary material for: DynamicSeq2SeqXGB for PM2.5 imputation in extremely sparse environmental monitoring networks
Source: PLoS One. 2025 Dec 11;20(12):e0338788. doi: 10.1371/journal.pone.0338788 (PMC12697988; doi:10.1371/journal.pone.0338788)
Supplement: S1 File — Fig S1. Monthly PM2.5 data completeness across five air quality monitoring stations in Pavlodar, Kazakhstan (May 2024 – July 2025), showing significant heterogeneity across stations and justifying full compression strategy adoption. Figs S2A-E. Data completeness heatmaps for each monitoring station, illustrating temporal patterns of missing and valid hourly observations with monthly completeness percentages. Fig S3. Scatter plots of true versus predicted PM2.5 concentrations for DynamicSeq2SeqXGB across all stations with color-coded gap lengths (5h-72h), demonstrating model performance variability by station and temporal scale. Fig S4. Beijing PM2.5 dataset characteristics including monthly completeness and concentration distributions from Guanyuan station (2016) used for external validation. Fig S5. Complex degradation analysis showing original time series, artificially degraded data (50% completeness), synthetic gap distribution, and DynamicSeq2SeqXGB reconstruction performance on Beijing dataset. Fig S6. Quantitative benchmarking comparison of imputation methods showing Mean Absolute Error (MAE) and Coefficient of Determination (R2) across all evaluated approaches. Fig S7. Summary of station-level optimal compression strategies across five gap lengths (5, 12, 24, 48, and 72 hours). Table S1. Complete benchmarking results for all imputation methods across stations and gap lengths using full compression strategy, including MAE, RMSE, R2, and MAPE metrics with standard deviations. Table S2. Complete benchmarking results for all imputation methods using selective compression strategy, providing parallel performance comparison for quality-filtered datasets. Table S3. Pearson correlation matrix of station characteristics and compression strategy performance metrics with calculation methodologies and significance indicators. Table S4. Beijing dataset experimental setup detailing synthetic degradation parameters, data partitions, and validation configuration for external [file pone.0338788.s001.docx]

**Figure S1. Monthly PM₂.₅ data completeness across five air quality monitoring stations in Pavlodar, Kazakhstan (May 2024 - July 2025).** Bar charts display monthly completeness percentages; the red dashed line indicates the 70% threshold. Average completeness for every station calculated as the arithmetic mean of monthly completeness percentages. Significant data heterogeneity (e.g., app_center 56.4% vs. app_metallurg 22.6% average monthly completeness) justifies the use of a full data compression strategy to maximize measurement preservation for gap-filling analysis.





**Figure S2A. Data completeness heatmap for app_center station (PM₂.₅ hourly measurements, May 2024 - July 2025).** Green = data available, red = missing data, white = outside monitoring period. Monthly completeness percentages shown in titles.





**Figure S2B. Data completeness heatmap for app_2pavlodar station (PM₂.₅ hourly measurements, May 2024 - July 2025).** Green = data available, red = missing data, white = outside monitoring period. Monthly completeness percentages shown in titles.





**Figure S2C. Data completeness heatmap for app_pspu station (PM₂.₅ hourly measurements, May 2024 - July 2025).** Green = data available, red = missing data, white = outside monitoring period. Monthly completeness percentages shown in titles.





**Figure S2D. Data completeness heatmap for app_metallurg station (PM₂.₅ hourly measurements, May 2024 - July 2025).** Green = data available, red = missing data, white = outside monitoring period. Monthly completeness percentages shown in titles.





**Figure S2E. Data completeness heatmap for app_zaton station (PM₂.₅ hourly measurements, May 2024 - July 2025).** Green = data available, red = missing data, white = outside monitoring period. Monthly completeness percentages shown in titles.


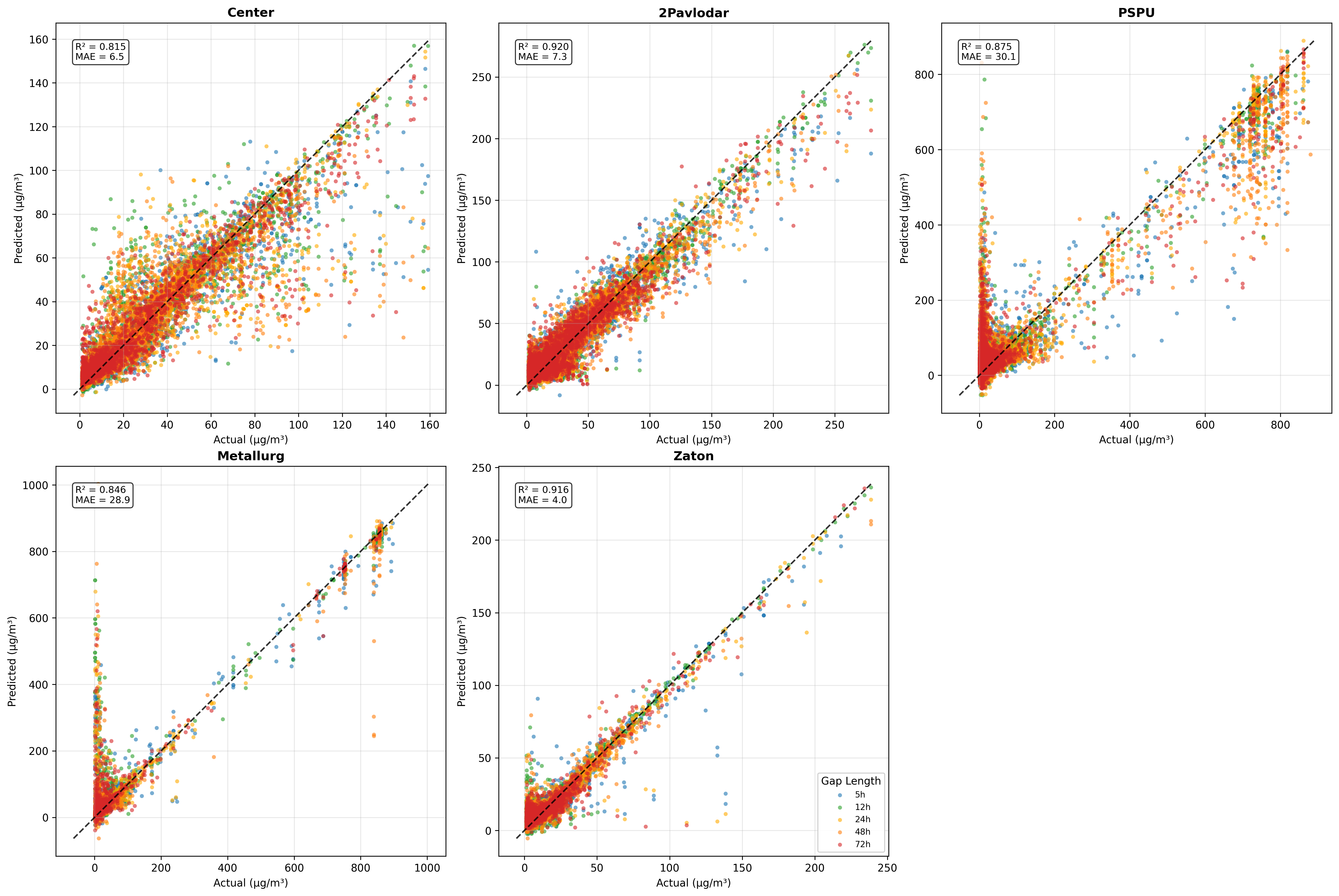


**Figure S3. Scatter plots of true versus predicted PM₂.₅ concentrations for the DynamicSeq2SeqXGB model across five monitoring stations using full data compression.** Each panel represents one monitoring station with color-coded points indicating different synthetic gap lengths: 5h (blue), 12h (green), 24h (yellow), 48h (orange), and 72h (red). The diagonal dashed line represents perfect prediction (y=x). Results are based on 10 independent runs with synthetic gaps created in dense data sequences. Model performance varies by station and gap length, with generally good agreement between predicted and observed values across all stations.

**Table S1 - Benchmarking of Imputation Methods Across All Stations and Gap Lengths (Data Preprocessing: Full Compression)**

| **Gap Length (h)** | **Method** | **MAE** | **RMSE** | **R2** | **MAPE** | **Station** |
| --- | --- | --- | --- | --- | --- | --- |
| 5 | DynamicSeq2SeqXGB | 5.635 ± 0.954 | 10.318 ± 2.492 | 0.849 ± 0.058 | 39.952 ± 7.548 | app_center |
| 5 | Forward Fill | 10.461 ± 1.069 | 18.493 ± 2.989 | 0.445 ± 0.145 | 69.685 ± 12.754 | app_center |
| 5 | Backward Fill | 10.314 ± 1.879 | 17.684 ± 3.847 | 0.496 ± 0.152 | 68.029 ± 13.192 | app_center |
| 5 | Linear Interpolation | 8.820 ± 0.000 | 16.365 ± 0.000 | 0.682 ± 0.000 | 40.510 ± 0.000 | app_center |
| 5 | Rolling Mean | 10.953 ± 1.617 | 17.059 ± 2.743 | 0.533 ± 0.104 | 96.868 ± 20.836 | app_center |
| 12 | DynamicSeq2SeqXGB | 5.843 ± 1.361 | 10.915 ± 3.042 | 0.843 ± 0.082 | 41.208 ± 15.646 | app_center |
| 12 | Forward Fill | 13.038 ± 2.341 | 21.045 ± 3.719 | 0.380 ± 0.261 | 97.861 ± 18.315 | app_center |
| 12 | Backward Fill | 12.524 ± 2.177 | 20.433 ± 4.284 | 0.417 ± 0.257 | 83.524 ± 32.331 | app_center |
| 12 | Linear Interpolation | 10.418 ± 1.340 | 16.997 ± 2.329 | 0.621 ± 0.124 | 63.108 ± 11.643 | app_center |
| 12 | Rolling Mean | 12.535 ± 0.952 | 18.812 ± 2.007 | 0.505 ± 0.211 | 101.744 ± 11.980 | app_center |
| 24 | DynamicSeq2SeqXGB | 6.777 ± 1.394 | 12.444 ± 2.416 | 0.769 ± 0.058 | 43.647 ± 7.405 | app_center |
| 24 | Forward Fill | 13.185 ± 2.000 | 20.129 ± 3.306 | 0.273 ± 0.344 | 109.266 ± 53.934 | app_center |
| 24 | Backward Fill | 14.803 ± 3.498 | 22.039 ± 5.222 | 0.170 ± 0.372 | 110.317 ± 41.731 | app_center |
| 24 | Linear Interpolation | 10.757 ± 2.035 | 16.096 ± 3.101 | 0.492 ± 0.193 | 74.698 ± 14.993 | app_center |
| 24 | Rolling Mean | 12.040 ± 1.376 | 17.204 ± 2.587 | 0.499 ± 0.147 | 119.472 ± 30.844 | app_center |
| 48 | DynamicSeq2SeqXGB | 7.942 ± 3.859 | 12.396 ± 7.266 | 0.743 ± 0.178 | 58.544 ± 10.300 | app_center |
| 48 | Forward Fill | 16.035 ± 5.020 | 23.355 ± 7.450 | -0.136 ± 0.544 | 157.144 ± 120.759 | app_center |
| 48 | Backward Fill | 13.831 ± 4.774 | 21.073 ± 6.279 | 0.075 ± 0.318 | 87.911 ± 33.125 | app_center |
| 48 | Linear Interpolation | 11.240 ± 3.836 | 17.016 ± 5.738 | 0.291 ± 0.209 | 78.749 ± 18.349 | app_center |
| 48 | Rolling Mean | 12.704 ± 3.210 | 18.491 ± 4.745 | 0.241 ± 0.324 | 118.537 ± 41.704 | app_center |
| 72 | DynamicSeq2SeqXGB | 6.576 ± 2.945 | 9.975 ± 5.283 | 0.693 ± 0.246 | 66.318 ± 43.082 | app_center |
| 72 | Forward Fill | 14.531 ± 7.883 | 20.438 ± 10.949 | -0.242 ± 0.397 | 142.132 ± 66.512 | app_center |
| 72 | Backward Fill | 15.820 ± 10.756 | 23.688 ± 15.955 | -0.675 ± 1.093 | 133.094 ± 98.426 | app_center |
| 72 | Linear Interpolation | 13.762 ± 8.399 | 20.359 ± 12.777 | -0.201 ± 0.678 | 131.058 ± 78.895 | app_center |
| 72 | Rolling Mean | 11.580 ± 5.303 | 17.209 ± 8.153 | 0.011 ± 0.379 | 112.034 ± 31.428 | app_center |
| 5 | DynamicSeq2SeqXGB | 7.390 ± 0.912 | 11.211 ± 1.811 | 0.905 ± 0.027 | 52.147 ± 6.932 | app_2pavlodar |
| 5 | Forward Fill | 16.790 ± 2.504 | 29.466 ± 5.627 | 0.307 ± 0.229 | 77.652 ± 10.896 | app_2pavlodar |
| 5 | Backward Fill | 16.350 ± 3.081 | 29.829 ± 7.217 | 0.302 ± 0.245 | 89.250 ± 22.812 | app_2pavlodar |
| 5 | Linear Interpolation | 8.528 ± 0.000 | 14.074 ± 0.000 | 0.765 ± 0.000 | 62.086 ± 0.000 | app_2pavlodar |
| 5 | Rolling Mean | 16.765 ± 2.336 | 27.667 ± 4.785 | 0.405 ± 0.114 | 113.852 ± 20.256 | app_2pavlodar |
| 12 | DynamicSeq2SeqXGB | 6.152 ± 0.605 | 8.625 ± 0.810 | 0.944 ± 0.025 | 56.580 ± 16.086 | app_2pavlodar |
| 12 | Forward Fill | 18.865 ± 4.567 | 31.297 ± 7.532 | 0.224 ± 0.153 | 101.316 ± 38.587 | app_2pavlodar |
| 12 | Backward Fill | 18.702 ± 4.599 | 31.038 ± 8.887 | 0.208 ± 0.319 | 118.836 ± 36.284 | app_2pavlodar |
| 12 | Linear Interpolation | 15.353 ± 4.050 | 26.632 ± 8.367 | 0.449 ± 0.152 | 83.352 ± 17.123 | app_2pavlodar |
| 12 | Rolling Mean | 16.458 ± 3.070 | 26.828 ± 6.644 | 0.420 ± 0.172 | 118.156 ± 25.632 | app_2pavlodar |
| 24 | DynamicSeq2SeqXGB | 6.786 ± 0.851 | 9.634 ± 1.469 | 0.907 ± 0.034 | 66.291 ± 9.439 | app_2pavlodar |
| 24 | Forward Fill | 23.141 ± 7.995 | 37.401 ± 12.397 | 0.053 ± 0.322 | 129.682 ± 57.052 | app_2pavlodar |
| 24 | Backward Fill | 24.393 ± 5.128 | 41.603 ± 9.651 | -0.287 ± 0.683 | 172.666 ± 100.121 | app_2pavlodar |
| 24 | Linear Interpolation | 21.645 ± 5.492 | 36.871 ± 9.140 | 0.146 ± 0.169 | 168.524 ± 64.272 | app_2pavlodar |
| 24 | Rolling Mean | 19.160 ± 4.709 | 29.583 ± 6.848 | 0.395 ± 0.148 | 126.936 ± 38.721 | app_2pavlodar |
| 48 | DynamicSeq2SeqXGB | 7.775 ± 0.971 | 10.714 ± 1.617 | 0.815 ± 0.245 | 74.147 ± 24.556 | app_2pavlodar |
| 48 | Forward Fill | 26.801 ± 9.232 | 44.327 ± 15.688 | -0.263 ± 0.507 | 138.089 ± 90.130 | app_2pavlodar |
| 48 | Backward Fill | 24.141 ± 7.523 | 40.058 ± 13.497 | -0.147 ± 0.686 | 175.723 ± 118.474 | app_2pavlodar |
| 48 | Linear Interpolation | 21.196 ± 5.908 | 35.557 ± 10.607 | 0.085 ± 0.327 | 102.302 ± 29.143 | app_2pavlodar |
| 48 | Rolling Mean | 20.583 ± 4.219 | 32.284 ± 6.847 | 0.184 ± 0.561 | 127.020 ± 25.414 | app_2pavlodar |
| 72 | DynamicSeq2SeqXGB | 8.515 ± 0.948 | 11.813 ± 1.516 | 0.766 ± 0.232 | 87.128 ± 36.802 | app_2pavlodar |
| 72 | Forward Fill | 25.153 ± 9.150 | 37.083 ± 11.803 | -0.036 ± 0.429 | 139.327 ± 110.535 | app_2pavlodar |
| 72 | Backward Fill | 25.719 ± 5.338 | 39.749 ± 8.949 | -0.197 ± 0.329 | 102.716 ± 36.655 | app_2pavlodar |
| 72 | Linear Interpolation | 24.440 ± 6.962 | 36.482 ± 9.112 | 0.146 ± 0.217 | 82.280 ± 8.187 | app_2pavlodar |
| 72 | Rolling Mean | 21.204 ± 5.932 | 33.617 ± 9.664 | 0.126 ± 0.381 | 120.017 ± 85.509 | app_2pavlodar |
| 5 | DynamicSeq2SeqXGB | 27.622 ± 3.767 | 55.091 ± 7.184 | 0.813 ± 0.194 | 238.361 ± 44.034 | app_pspu |
| 5 | Forward Fill | 53.077 ± 14.306 | 157.790 ± 26.016 | 0.015 ± 0.479 | 206.328 ± 145.990 | app_pspu |
| 5 | Backward Fill | 53.572 ± 18.276 | 154.106 ± 39.939 | 0.088 ± 0.413 | 329.292 ± 193.575 | app_pspu |
| 5 | Rolling Mean | 68.764 ± 10.241 | 146.809 ± 15.727 | 0.190 ± 0.155 | 510.761 ± 181.419 | app_pspu |
| 12 | DynamicSeq2SeqXGB | 24.848 ± 5.472 | 49.157 ± 19.110 | 0.842 ± 0.165 | 266.325 ± 90.450 | app_pspu |
| 12 | Forward Fill | 57.752 ± 22.371 | 167.413 ± 42.616 | -0.475 ± 0.639 | 422.545 ± 324.392 | app_pspu |
| 12 | Backward Fill | 56.409 ± 16.652 | 157.455 ± 35.550 | -0.255 ± 0.294 | 372.232 ± 229.567 | app_pspu |
| 12 | Linear Interpolation | 49.106 ± 14.232 | 139.571 ± 39.557 | -0.116 ± 0.603 | 201.380 ± 151.977 | app_pspu |
| 12 | Rolling Mean | 57.191 ± 13.151 | 126.308 ± 25.972 | 0.188 ± 0.194 | 470.871 ± 148.958 | app_pspu |
| 24 | DynamicSeq2SeqXGB | 27.413 ± 5.244 | 50.712 ± 12.135 | 0.772 ± 0.281 | 331.263 ± 148.676 | app_pspu |
| 24 | Forward Fill | 73.709 ± 28.138 | 185.281 ± 52.776 | -1.081 ± 2.465 | 384.524 ± 296.631 | app_pspu |
| 24 | Backward Fill | 70.745 ± 24.058 | 186.427 ± 34.829 | -0.991 ± 1.761 | 406.453 ± 347.697 | app_pspu |
| 24 | Linear Interpolation | 62.143 ± 18.483 | 161.677 ± 33.101 | -1.200 ± 2.436 | 386.624 ± 156.986 | app_pspu |
| 24 | Rolling Mean | 70.121 ± 15.691 | 145.278 ± 32.137 | 0.024 ± 0.340 | 511.569 ± 226.041 | app_pspu |
| 48 | DynamicSeq2SeqXGB | 39.199 ± 12.607 | 70.773 ± 28.868 | -11.818 ± 38.097 | 427.479 ± 257.008 | app_pspu |
| 48 | Forward Fill | 70.639 ± 57.330 | 156.991 ± 108.325 | -0.291 ± 0.322 | 394.763 ± 332.788 | app_pspu |
| 48 | Backward Fill | 57.679 ± 44.226 | 138.787 ± 89.657 | -0.114 ± 0.150 | 426.185 ± 617.886 | app_pspu |
| 48 | Linear Interpolation | 30.558 ± 21.656 | 81.688 ± 55.417 | 0.047 ± 0.057 | 177.717 ± 125.793 | app_pspu |
| 48 | Rolling Mean | 65.027 ± 48.850 | 126.540 ± 73.428 | -0.077 ± 0.353 | 436.203 ± 323.823 | app_pspu |
| 72 | DynamicSeq2SeqXGB | 31.207 ± 11.359 | 53.398 ± 23.587 | -0.284 ± 2.179 | 415.323 ± 238.560 | app_pspu |
| 72 | Forward Fill | 49.423 ± 64.413 | 103.596 ± 106.165 | -0.106 ± 0.387 | 196.755 ± 258.325 | app_pspu |
| 72 | Backward Fill | 39.116 ± 33.413 | 93.302 ± 78.726 | -0.046 ± 0.172 | 366.313 ± 750.526 | app_pspu |
| 72 | Linear Interpolation | 47.231 ± 51.771 | 102.522 ± 86.741 | 0.033 ± 0.220 | 324.478 ± 571.185 | app_pspu |
| 72 | Rolling Mean | 50.740 ± 39.175 | 86.670 ± 66.509 | 0.031 ± 0.282 | 410.037 ± 352.088 | app_pspu |
| 5 | DynamicSeq2SeqXGB | 25.614 ± 8.160 | 55.659 ± 22.614 | 0.820 ± 0.306 | 362.907 ± 288.540 | app_metallurg |
| 5 | Forward Fill | 43.981 ± 16.728 | 126.777 ± 42.528 | -3.031 ± 9.462 | 320.864 ± 405.323 | app_metallurg |
| 5 | Backward Fill | 55.726 ± 31.342 | 152.597 ± 66.659 | -1.979 ± 7.063 | 252.020 ± 338.075 | app_metallurg |
| 5 | Linear Interpolation | 46.818 ± 8.305 | 120.085 ± 8.419 | -3.280 ± 6.478 | 255.363 ± 237.545 | app_metallurg |
| 5 | Rolling Mean | 68.114 ± 25.675 | 135.791 ± 47.667 | 0.142 ± 0.914 | 495.290 ± 191.617 | app_metallurg |
| 12 | DynamicSeq2SeqXGB | 31.142 ± 19.807 | 68.957 ± 52.557 | -7.711 ± 16.880 | 696.831 ± 954.777 | app_metallurg |
| 12 | Forward Fill | 51.739 ± 41.644 | 140.993 ± 86.720 | -0.016 ± 0.764 | 430.055 ± 876.535 | app_metallurg |
| 12 | Backward Fill | 56.021 ± 33.456 | 139.875 ± 77.955 | 0.062 ± 0.655 | 346.869 ± 393.253 | app_metallurg |
| 12 | Linear Interpolation | 42.283 ± 24.223 | 116.253 ± 59.464 | 0.437 ± 0.260 | 239.263 ± 198.882 | app_metallurg |
| 12 | Rolling Mean | 61.768 ± 24.849 | 126.403 ± 51.925 | 0.121 ± 0.669 | 378.060 ± 202.444 | app_metallurg |
| 24 | DynamicSeq2SeqXGB | 27.179 ± 26.483 | 49.996 ± 53.398 | -12.541 ± 25.920 | 493.225 ± 757.783 | app_metallurg |
| 24 | Forward Fill | 62.834 ± 53.939 | 137.969 ± 114.195 | -0.183 ± 0.542 | 204.765 ± 213.134 | app_metallurg |
| 24 | Backward Fill | 62.470 ± 61.064 | 136.269 ± 109.942 | -0.540 ± 1.341 | 388.232 ± 814.754 | app_metallurg |
| 24 | Linear Interpolation | 42.567 ± 31.756 | 97.816 ± 76.478 | 0.180 ± 0.345 | 145.808 ± 52.245 | app_metallurg |
| 24 | Rolling Mean | 54.813 ± 35.312 | 105.668 ± 73.472 | 0.120 ± 0.262 | 313.131 ± 224.171 | app_metallurg |
| 48 | DynamicSeq2SeqXGB | 29.705 ± 37.168 | 50.388 ± 65.422 | -75.410 ± 228.147 | 275.831 ± 405.955 | app_metallurg |
| 48 | Forward Fill | 71.928 ± 92.876 | 141.076 ± 138.163 | -0.372 ± 1.088 | 283.370 ± 536.529 | app_metallurg |
| 48 | Backward Fill | 94.757 ± 107.632 | 156.578 ± 139.628 | -1.671 ± 2.541 | 440.820 ± 587.682 | app_metallurg |
| 48 | Linear Interpolation | 64.997 ± 60.236 | 115.786 ± 86.078 | -0.263 ± 0.627 | 279.606 ± 257.341 | app_metallurg |
| 48 | Rolling Mean | 78.928 ± 77.642 | 127.909 ± 98.989 | -0.501 ± 0.929 | 471.876 ± 513.678 | app_metallurg |
| 72 | DynamicSeq2SeqXGB | 32.231 ± 42.850 | 45.216 ± 59.157 | -210.686 ± 628.854 | 554.132 ± 1276.098 | app_metallurg |
| 72 | Forward Fill | 184.347 ± 158.778 | 259.372 ± 194.377 | -0.541 ± 0.404 | 579.348 ± 877.166 | app_metallurg |
| 72 | Backward Fill | 201.612 ± 201.253 | 280.648 ± 233.027 | -0.587 ± 0.838 | 265.503 ± 489.751 | app_metallurg |
| 72 | Linear Interpolation | 168.337 ± 155.961 | 237.215 ± 186.049 | -0.245 ± 0.469 | 332.247 ± 492.279 | app_metallurg |
| 72 | Rolling Mean | 197.803 ± 159.162 | 245.949 ± 176.909 | -0.457 ± 0.710 | 789.042 ± 816.703 | app_metallurg |
| 5 | DynamicSeq2SeqXGB | 4.077 ± 1.064 | 7.411 ± 3.517 | 0.861 ± 0.137 | 58.477 ± 9.892 | app_zaton |
| 5 | Forward Fill | 9.964 ± 3.407 | 21.580 ± 9.728 | 0.117 ± 0.650 | 93.768 ± 47.581 | app_zaton |
| 5 | Backward Fill | 9.570 ± 3.285 | 19.317 ± 9.068 | 0.387 ± 0.278 | 81.010 ± 21.500 | app_zaton |
| 5 | Linear Interpolation | 5.396 ± 0.000 | 8.819 ± 0.000 | 0.629 ± 0.000 | 54.061 ± 0.000 | app_zaton |
| 5 | Rolling Mean | 9.916 ± 2.566 | 18.550 ± 7.522 | 0.429 ± 0.194 | 120.424 ± 21.524 | app_zaton |
| 12 | DynamicSeq2SeqXGB | 3.522 ± 0.599 | 6.050 ± 1.503 | 0.679 ± 0.461 | 86.329 ± 36.577 | app_zaton |
| 12 | Forward Fill | 9.335 ± 2.606 | 16.956 ± 7.138 | 0.263 ± 0.309 | 82.746 ± 16.339 | app_zaton |
| 12 | Backward Fill | 10.686 ± 3.288 | 18.870 ± 8.437 | 0.113 ± 0.361 | 100.798 ± 25.522 | app_zaton |
| 12 | Linear Interpolation | 8.784 ± 2.897 | 17.547 ± 7.700 | 0.291 ± 0.133 | 64.392 ± 12.613 | app_zaton |
| 12 | Rolling Mean | 9.338 ± 1.896 | 16.013 ± 5.638 | 0.293 ± 0.374 | 117.392 ± 30.078 | app_zaton |
| 24 | DynamicSeq2SeqXGB | 3.672 ± 1.109 | 6.632 ± 4.213 | 0.853 ± 0.175 | 54.587 ± 17.025 | app_zaton |
| 24 | Forward Fill | 10.544 ± 3.814 | 17.782 ± 8.894 | -0.263 ± 0.724 | 127.106 ± 49.840 | app_zaton |
| 24 | Backward Fill | 10.365 ± 3.359 | 17.234 ± 6.141 | -0.538 ± 1.569 | 147.044 ± 102.740 | app_zaton |
| 24 | Linear Interpolation | 9.177 ± 2.613 | 16.232 ± 6.436 | -0.126 ± 0.898 | 121.654 ± 68.773 | app_zaton |
| 24 | Rolling Mean | 9.905 ± 2.459 | 15.547 ± 5.854 | -0.054 ± 0.671 | 159.572 ± 63.825 | app_zaton |
| 48 | DynamicSeq2SeqXGB | 4.504 ± 1.205 | 6.434 ± 2.132 | 0.695 ± 0.432 | 98.064 ± 32.893 | app_zaton |
| 48 | Forward Fill | 10.026 ± 5.197 | 16.422 ± 8.382 | -0.670 ± 1.160 | 154.487 ± 181.649 | app_zaton |
| 48 | Backward Fill | 10.097 ± 4.024 | 15.269 ± 6.130 | -0.463 ± 0.838 | 171.205 ± 85.395 | app_zaton |
| 48 | Linear Interpolation | 8.800 ± 3.078 | 14.411 ± 6.060 | -0.227 ± 0.451 | 144.025 ± 86.934 | app_zaton |
| 48 | Rolling Mean | 8.728 ± 3.242 | 13.974 ± 6.729 | -0.039 ± 0.182 | 144.975 ± 56.951 | app_zaton |
| 72 | DynamicSeq2SeqXGB | 4.518 ± 1.740 | 6.544 ± 3.403 | 0.662 ± 0.326 | 73.660 ± 54.109 | app_zaton |
| 72 | Forward Fill | 12.731 ± 6.975 | 20.284 ± 11.370 | -2.015 ± 5.205 | 116.950 ± 102.764 | app_zaton |
| 72 | Backward Fill | 15.426 ± 11.357 | 22.056 ± 15.691 | -1.107 ± 1.816 | 147.588 ± 94.617 | app_zaton |
| 72 | Linear Interpolation | 10.824 ± 5.350 | 18.114 ± 11.026 | -0.909 ± 2.150 | 113.076 ± 72.641 | app_zaton |
| 72 | Rolling Mean | 11.670 ± 7.001 | 17.568 ± 11.307 | -0.179 ± 0.350 | 156.633 ± 109.604 | app_zaton |

**Table S2 - Benchmarking of Imputation Methods Across All Stations and Gap Lengths (Data Preprocessing: Selective Compression)**

| **Gap Length (h)** | **Method** | **MAE** | **RMSE** | **R2** | **MAPE** | **Station** |
| --- | --- | --- | --- | --- | --- | --- |
| 5 | DynamicSeq2SeqXGB | 5.553 ± 0.726 | 9.844 ± 1.933 | 0.872 ± 0.037 | 31.968 ± 4.657 | app_center |
| 5 | Forward Fill | 10.051 ± 1.422 | 17.483 ± 2.739 | 0.612 ± 0.127 | 52.655 ± 11.643 | app_center |
| 5 | Backward Fill | 10.435 ± 1.207 | 18.095 ± 2.468 | 0.589 ± 0.101 | 52.669 ± 10.971 | app_center |
| 5 | Linear Interpolation | 6.636 ± 0.000 | 10.732 ± 0.000 | 0.851 ± 0.000 | 41.814 ± 0.000 | app_center |
| 5 | Rolling Mean | 11.621 ± 0.724 | 17.936 ± 1.027 | 0.599 ± 0.058 | 81.571 ± 12.658 | app_center |
| 12 | DynamicSeq2SeqXGB | 5.736 ± 1.351 | 12.185 ± 2.966 | 0.752 ± 0.120 | 47.425 ± 44.377 | app_center |
| 12 | Forward Fill | 13.812 ± 4.292 | 21.580 ± 6.064 | 0.420 ± 0.210 | 74.248 ± 17.179 | app_center |
| 12 | Backward Fill | 14.546 ± 4.684 | 22.166 ± 6.685 | 0.369 ± 0.298 | 82.448 ± 27.019 | app_center |
| 12 | Linear Interpolation | 12.455 ± 2.815 | 19.635 ± 4.203 | 0.517 ± 0.115 | 52.748 ± 12.019 | app_center |
| 12 | Rolling Mean | 13.576 ± 3.469 | 19.619 ± 4.699 | 0.527 ± 0.117 | 99.773 ± 27.801 | app_center |
| 24 | DynamicSeq2SeqXGB | 7.630 ± 1.664 | 14.551 ± 3.098 | 0.675 ± 0.169 | 48.567 ± 28.250 | app_center |
| 24 | Forward Fill | 16.552 ± 2.888 | 24.215 ± 5.594 | 0.157 ± 0.344 | 122.166 ± 44.100 | app_center |
| 24 | Backward Fill | 14.777 ± 2.537 | 22.501 ± 3.476 | 0.168 ± 0.680 | 88.261 ± 40.058 | app_center |
| 24 | Linear Interpolation | 12.874 ± 1.976 | 19.474 ± 3.676 | 0.435 ± 0.356 | 77.435 ± 27.933 | app_center |
| 24 | Rolling Mean | 13.886 ± 3.249 | 20.070 ± 3.861 | 0.378 ± 0.337 | 113.637 ± 36.690 | app_center |
| 48 | DynamicSeq2SeqXGB | 6.277 ± 1.971 | 9.637 ± 3.608 | 0.788 ± 0.265 | 36.634 ± 10.678 | app_center |
| 48 | Forward Fill | 19.486 ± 4.619 | 27.963 ± 6.431 | -0.295 ± 0.525 | 108.727 ± 63.928 | app_center |
| 48 | Backward Fill | 18.906 ± 7.727 | 25.500 ± 9.020 | 0.029 ± 0.359 | 110.952 ± 56.888 | app_center |
| 48 | Linear Interpolation | 15.918 ± 6.275 | 22.421 ± 8.388 | 0.247 ± 0.207 | 85.752 ± 40.658 | app_center |
| 48 | Rolling Mean | 16.699 ± 4.236 | 22.984 ± 5.780 | 0.192 ± 0.187 | 105.736 ± 49.694 | app_center |
| 72 | DynamicSeq2SeqXGB | 10.255 ± 5.063 | 15.243 ± 7.689 | 0.401 ± 0.546 | 87.743 ± 66.856 | app_center |
| 72 | Forward Fill | 20.599 ± 6.484 | 29.209 ± 8.828 | -0.154 ± 0.444 | 145.372 ± 99.992 | app_center |
| 72 | Backward Fill | 20.155 ± 8.641 | 28.347 ± 9.326 | -0.048 ± 0.377 | 131.578 ± 53.093 | app_center |
| 72 | Linear Interpolation | 17.172 ± 7.454 | 25.019 ± 9.190 | 0.120 ± 0.295 | 127.154 ± 49.658 | app_center |
| 72 | Rolling Mean | 17.301 ± 6.176 | 24.873 ± 7.085 | 0.183 ± 0.263 | 133.216 ± 55.440 | app_center |
| 5 | DynamicSeq2SeqXGB | 7.977 ± 0.645 | 11.371 ± 1.400 | 0.925 ± 0.029 | 56.881 ± 21.992 | app_2pavlodar |
| 5 | Forward Fill | 20.527 ± 3.455 | 35.484 ± 5.894 | 0.196 ± 0.118 | 72.764 ± 19.317 | app_2pavlodar |
| 5 | Backward Fill | 19.776 ± 5.131 | 34.234 ± 10.057 | 0.248 ± 0.288 | 73.369 ± 15.388 | app_2pavlodar |
| 5 | Linear Interpolation | 15.606 ± 0.000 | 24.314 ± 0.000 | 0.568 ± 0.000 | 52.953 ± 0.000 | app_2pavlodar |
| 5 | Rolling Mean | 20.292 ± 3.206 | 31.559 ± 5.943 | 0.356 ± 0.154 | 96.558 ± 19.154 | app_2pavlodar |
| 12 | DynamicSeq2SeqXGB | 7.353 ± 1.148 | 11.474 ± 2.224 | 0.893 ± 0.082 | 81.387 ± 34.817 | app_2pavlodar |
| 12 | Forward Fill | 30.343 ± 7.975 | 48.721 ± 11.072 | -0.432 ± 0.992 | 100.668 ± 62.793 | app_2pavlodar |
| 12 | Backward Fill | 28.618 ± 9.777 | 44.648 ± 17.074 | -0.105 ± 0.613 | 157.977 ± 159.854 | app_2pavlodar |
| 12 | Linear Interpolation | 22.193 ± 5.673 | 35.491 ± 9.094 | 0.246 ± 0.318 | 95.522 ± 58.864 | app_2pavlodar |
| 12 | Rolling Mean | 23.404 ± 5.841 | 35.464 ± 8.519 | 0.330 ± 0.120 | 96.574 ± 32.946 | app_2pavlodar |
| 24 | DynamicSeq2SeqXGB | 9.301 ± 2.385 | 14.480 ± 4.077 | 0.731 ± 0.278 | 100.740 ± 66.533 | app_2pavlodar |
| 24 | Forward Fill | 27.003 ± 4.702 | 40.293 ± 8.234 | -0.328 ± 0.731 | 143.582 ± 65.402 | app_2pavlodar |
| 24 | Backward Fill | 24.575 ± 7.056 | 38.004 ± 10.447 | -0.137 ± 0.714 | 110.378 ± 63.592 | app_2pavlodar |
| 24 | Linear Interpolation | 21.808 ± 3.694 | 32.553 ± 4.627 | 0.200 ± 0.274 | 118.542 ± 29.810 | app_2pavlodar |
| 24 | Rolling Mean | 19.269 ± 4.138 | 28.674 ± 6.579 | 0.396 ± 0.180 | 107.632 ± 35.055 | app_2pavlodar |
| 48 | DynamicSeq2SeqXGB | 12.003 ± 5.721 | 16.910 ± 7.741 | -0.117 ± 2.593 | 127.118 ± 162.738 | app_2pavlodar |
| 48 | Forward Fill | 33.443 ± 14.606 | 47.008 ± 18.488 | -0.598 ± 0.681 | 170.623 ± 180.596 | app_2pavlodar |
| 48 | Backward Fill | 32.647 ± 23.874 | 45.808 ± 29.546 | -0.819 ± 2.166 | 118.934 ± 70.463 | app_2pavlodar |
| 48 | Linear Interpolation | 29.497 ± 12.847 | 42.394 ± 15.518 | -0.242 ± 0.591 | 120.235 ± 103.085 | app_2pavlodar |
| 48 | Rolling Mean | 25.009 ± 8.453 | 35.879 ± 11.866 | 0.029 ± 0.355 | 109.523 ± 36.692 | app_2pavlodar |
| 72 | DynamicSeq2SeqXGB | 12.184 ± 4.660 | 17.614 ± 8.523 | 0.758 ± 0.160 | 63.722 ± 59.680 | app_2pavlodar |
| 72 | Forward Fill | 23.467 ± 8.804 | 35.119 ± 14.497 | -0.411 ± 0.591 | 106.471 ± 59.648 | app_2pavlodar |
| 72 | Backward Fill | 22.597 ± 13.896 | 33.305 ± 19.827 | -0.170 ± 0.686 | 99.815 ± 87.625 | app_2pavlodar |
| 72 | Linear Interpolation | 20.804 ± 8.518 | 31.124 ± 13.514 | -0.072 ± 0.301 | 98.840 ± 67.670 | app_2pavlodar |
| 72 | Rolling Mean | 20.477 ± 5.183 | 30.962 ± 9.951 | -0.245 ± 0.756 | 92.663 ± 27.803 | app_2pavlodar |
| 5 | DynamicSeq2SeqXGB | 23.554 ± 5.209 | 46.179 ± 10.851 | 0.757 ± 0.514 | 194.162 ± 55.041 | app_pspu |
| 5 | Forward Fill | 52.207 ± 26.087 | 148.771 ± 52.968 | -0.304 ± 0.794 | 275.858 ± 293.584 | app_pspu |
| 5 | Backward Fill | 49.967 ± 14.790 | 143.363 ± 34.708 | -0.368 ± 0.926 | 324.857 ± 232.893 | app_pspu |
| 5 | Linear Interpolation | 34.457 ± 0.000 | 125.532 ± 0.000 | -0.026 ± 0.000 | 163.918 ± 0.000 | app_pspu |
| 5 | Rolling Mean | 69.248 ± 20.768 | 134.837 ± 34.553 | -0.165 ± 0.777 | 619.960 ± 394.289 | app_pspu |
| 12 | DynamicSeq2SeqXGB | 21.394 ± 5.767 | 38.495 ± 15.518 | 0.878 ± 0.133 | 286.903 ± 223.201 | app_pspu |
| 12 | Forward Fill | 75.963 ± 35.065 | 182.571 ± 62.700 | -1.320 ± 1.553 | 891.047 ± 545.663 | app_pspu |
| 12 | Backward Fill | 72.817 ± 44.162 | 180.063 ± 73.092 | -1.976 ± 4.577 | 706.424 ± 710.725 | app_pspu |
| 12 | Linear Interpolation | 60.871 ± 35.244 | 146.595 ± 51.665 | -1.504 ± 3.084 | 762.040 ± 602.285 | app_pspu |
| 12 | Rolling Mean | 73.803 ± 29.020 | 140.771 ± 40.622 | -0.474 ± 1.448 | 800.773 ± 406.354 | app_pspu |
| 24 | DynamicSeq2SeqXGB | 19.433 ± 4.259 | 31.824 ± 6.047 | 0.791 ± 0.279 | 215.342 ± 65.319 | app_pspu |
| 24 | Forward Fill | 96.290 ± 40.342 | 222.683 ± 71.158 | -0.989 ± 1.160 | 700.308 ± 525.438 | app_pspu |
| 24 | Backward Fill | 72.975 ± 22.661 | 179.594 ± 48.858 | -0.236 ± 0.284 | 224.579 ± 190.002 | app_pspu |
| 24 | Linear Interpolation | 70.863 ± 29.333 | 156.794 ± 54.055 | 0.029 ± 0.241 | 408.656 ± 234.504 | app_pspu |
| 24 | Rolling Mean | 79.549 ± 27.862 | 158.280 ± 46.490 | 0.065 ± 0.157 | 426.424 ± 160.539 | app_pspu |
| 48 | DynamicSeq2SeqXGB | 32.571 ± 13.114 | 63.240 ± 30.772 | -0.506 ± 3.883 | 220.989 ± 83.160 | app_pspu |
| 48 | Forward Fill | 75.065 ± 62.721 | 152.843 ± 89.505 | -0.841 ± 1.591 | 773.716 ± 1226.264 | app_pspu |
| 48 | Backward Fill | 94.808 ± 82.335 | 189.439 ± 134.985 | -1.678 ± 4.254 | 375.224 ± 491.166 | app_pspu |
| 48 | Linear Interpolation | 59.774 ± 37.764 | 130.613 ± 72.558 | -0.652 ± 1.303 | 370.052 ± 336.130 | app_pspu |
| 48 | Rolling Mean | 75.444 ± 56.448 | 139.848 ± 78.041 | -2.622 ± 7.916 | 469.909 ± 417.247 | app_pspu |
| 72 | DynamicSeq2SeqXGB | 26.521 ± 10.793 | 46.706 ± 23.935 | -1.034 ± 2.959 | 306.279 ± 169.203 | app_pspu |
| 72 | Forward Fill | 63.985 ± 52.250 | 138.569 ± 80.577 | -0.090 ± 0.210 | 311.511 ± 374.563 | app_pspu |
| 72 | Backward Fill | 77.146 ± 73.430 | 165.948 ± 116.377 | -0.431 ± 0.658 | 428.836 ± 829.574 | app_pspu |
| 72 | Linear Interpolation | 69.592 ± 64.383 | 150.678 ± 97.480 | -0.165 ± 0.344 | 333.843 ± 487.814 | app_pspu |
| 72 | Rolling Mean | 72.319 ± 46.448 | 137.719 ± 79.700 | -0.152 ± 0.467 | 462.990 ± 333.101 | app_pspu |
| 5 | DynamicSeq2SeqXGB | 3.023 ± 1.155 | 6.602 ± 4.306 | 0.635 ± 0.208 | 40.012 ± 16.045 | app_zaton |
| 5 | Forward Fill | 7.675 ± 2.431 | 12.508 ± 4.504 | -0.909 ± 1.217 | 88.334 ± 32.632 | app_zaton |
| 5 | Backward Fill | 7.336 ± 1.435 | 12.631 ± 4.077 | -1.540 ± 3.004 | 108.420 ± 66.650 | app_zaton |
| 5 | Linear Interpolation | 5.979 ± 0.292 | 10.679 ± 0.721 | 0.063 ± 0.066 | 56.247 ± 8.622 | app_zaton |
| 5 | Rolling Mean | 6.990 ± 1.764 | 9.947 ± 4.476 | -0.012 ± 0.184 | 113.975 ± 29.374 | app_zaton |
| 12 | DynamicSeq2SeqXGB | 3.949 ± 1.811 | 10.074 ± 5.928 | 0.431 ± 0.206 | 59.984 ± 35.745 | app_zaton |
| 12 | Forward Fill | 8.756 ± 2.667 | 14.883 ± 5.865 | -2.246 ± 4.461 | 120.438 ± 42.532 | app_zaton |
| 12 | Backward Fill | 9.311 ± 6.815 | 16.800 ± 15.441 | -2.621 ± 6.880 | 113.401 ± 74.203 | app_zaton |
| 12 | Linear Interpolation | 8.456 ± 4.383 | 16.626 ± 9.629 | -1.689 ± 2.868 | 90.668 ± 33.742 | app_zaton |
| 12 | Rolling Mean | 7.719 ± 2.250 | 11.562 ± 5.558 | -0.214 ± 0.577 | 135.757 ± 35.302 | app_zaton |
| 24 | DynamicSeq2SeqXGB | 5.445 ± 3.381 | 12.238 ± 10.270 | 0.375 ± 0.352 | 90.721 ± 84.989 | app_zaton |
| 24 | Forward Fill | 11.660 ± 6.231 | 18.641 ± 11.584 | -1.096 ± 1.939 | 158.491 ± 133.427 | app_zaton |
| 24 | Backward Fill | 10.040 ± 3.642 | 16.103 ± 8.755 | -0.368 ± 0.394 | 104.071 ± 49.642 | app_zaton |
| 24 | Linear Interpolation | 9.497 ± 4.737 | 15.749 ± 10.167 | -0.253 ± 0.727 | 111.114 ± 79.676 | app_zaton |
| 24 | Rolling Mean | 9.108 ± 3.186 | 14.624 ± 9.112 | -0.048 ± 0.174 | 124.690 ± 37.796 | app_zaton |
| 48 | DynamicSeq2SeqXGB | 3.309 ± 4.427 | 6.789 ± 11.597 | 0.773 ± 0.381 | 43.142 ± 51.007 | app_zaton |
| 48 | Forward Fill | 10.977 ± 9.824 | 14.754 ± 13.083 | -1.635 ± 2.066 | 235.513 ± 298.716 | app_zaton |
| 48 | Backward Fill | 8.075 ± 3.048 | 10.945 ± 3.603 | -0.753 ± 0.710 | 155.001 ± 110.958 | app_zaton |
| 48 | Linear Interpolation | 8.696 ± 5.902 | 11.997 ± 7.400 | -0.843 ± 0.871 | 173.264 ± 169.840 | app_zaton |
| 48 | Rolling Mean | 7.348 ± 2.368 | 9.526 ± 3.449 | -0.357 ± 0.773 | 148.743 ± 98.087 | app_zaton |
| 72 | DynamicSeq2SeqXGB | 6.033 ± 5.473 | 8.519 ± 8.966 | 0.377 ± 0.561 | 92.493 ± 71.577 | app_zaton |
| 72 | Forward Fill | 11.961 ± 6.325 | 17.510 ± 12.573 | -1.068 ± 1.253 | 163.386 ± 124.891 | app_zaton |
| 72 | Backward Fill | 12.308 ± 9.575 | 16.433 ± 12.873 | -0.471 ± 0.432 | 147.555 ± 131.508 | app_zaton |

**Table S3. Pearson correlation matrix of station characteristics and compression strategy performance metrics**

| **Variable** | **1** | **2** | **3** | **4** | **5** | **6** | **7** | **8** | **9** |
| --- | --- | --- | --- | --- | --- | --- | --- | --- | --- |
| 1. Improvement_Full_vs_Selective_% | 1 | **-0.859** | **-0.78** | -0.087 | **0.886** | **-0.725** | **-0.86** | 0.085 | -0.109 |
| 2. Full_Compression_MAE | **-0.859** | 1 | ***0.99*** | 0.465 | **-0.858** | ***0.975*** | ***0.997*** | -0.461 | 0.299 |
| 3. Selective_Compression_MAE | **-0.78** | ***0.99*** | 1 | 0.555 | **-0.803** | ***0.996*** | ***0.984*** | -0.55 | 0.322 |
| 4. Completeness_% | -0.087 | 0.465 | 0.555 | 1 | 0.049 | 0.608 | 0.406 | ***-1*** | -0.267 |
| 5. Homogeneity | **0.886** | **-0.858** | **-0.803** | 0.049 | 1 | **-0.76** | **-0.893** | -0.055 | -0.557 |
| 6. Mean_PM25 | **-0.725** | ***0.975*** | ***0.996*** | 0.608 | **-0.76** | 1 | ***0.967*** | -0.602 | 0.327 |
| 7. Std_PM25 | **-0.86** | ***0.997*** | ***0.984*** | 0.406 | **-0.893** | ***0.967*** | 1 | -0.401 | 0.371 |
| 8. Missing_Count | 0.085 | -0.461 | -0.55 | ***-1*** | -0.055 | -0.602 | -0.401 | 1 | 0.277 |
| 9. Total_Records | -0.109 | 0.299 | 0.322 | -0.267 | -0.557 | 0.327 | 0.371 | 0.277 | 1 |

**Bold** indicates strong correlations (|r| > 0.7), *italic* indicates statistically significant correlations (p < 0.05), and ***bold italic*** indicates correlations meeting both criteria.

Calculation methods:

- Improvement_Full_vs_Selective_%: ((Selective_MAE - Full_MAE) / Selective_MAE) × 100
- Full_Compression_MAE: Mean Absolute Error of DynamicSeq2SeqXGB on complete dataset after outlier removal
- Selective_Compression_MAE: Mean Absolute Error of DynamicSeq2SeqXGB on dataset filtered to 70% completeness threshold
- Completeness_%: (Non-missing PM2.5 values / Total possible values) × 100
- Homogeneity: 1 / (1 + CV), where CV = Std_PM25 / Mean_PM25
- Mean_PM25: Arithmetic mean of PM2.5 concentrations (µg/m³) from cleaned data
- Std_PM25: Standard deviation of PM2.5 concentrations (µg/m³) from cleaned data
- Missing_Count: Total_Records × (100 - Completeness_%) / 100
- Total_Records: Period_days × 24 (estimated hourly records)

**Table S4.** **Beijing dataset characteristics and experimental setup.** Overview of the Beijing Guanyuan PM₂.₅ monitoring data (2016) utilized for external validation, detailing dataset properties, synthetic degradation parameters, and resulting data partitions for imputation model evaluation under controlled missingness conditions.

| **Aspect** | **Parameter** | **Value** | **Notes** |
| --- | --- | --- | --- |
| Dataset | Time period | 2016 year | Full calendar year |
| Dataset | Frequency | Hourly measurements | 8,784 observations |
| Dataset | Initial completeness | 98.41% | 140 gaps in original |
| Degradation | Target completeness | 54.64% | After applying synthetic gaps |
| Degradation | Total artificial gaps | 3,844 | Sum of all gap types |
| Degradation | Gap sizes | 773, 383, 360, 193, 180, 95, 96, 90, 48 h | 9 structured gaps based on app_pspu |
| Degradation | Random missing points | 2,408 | Uniformly distributed |
| Processing | Training data | 4,800 records | Dense sequences |
| Processing | Test data | 3,844 records | Artificial missing observations |


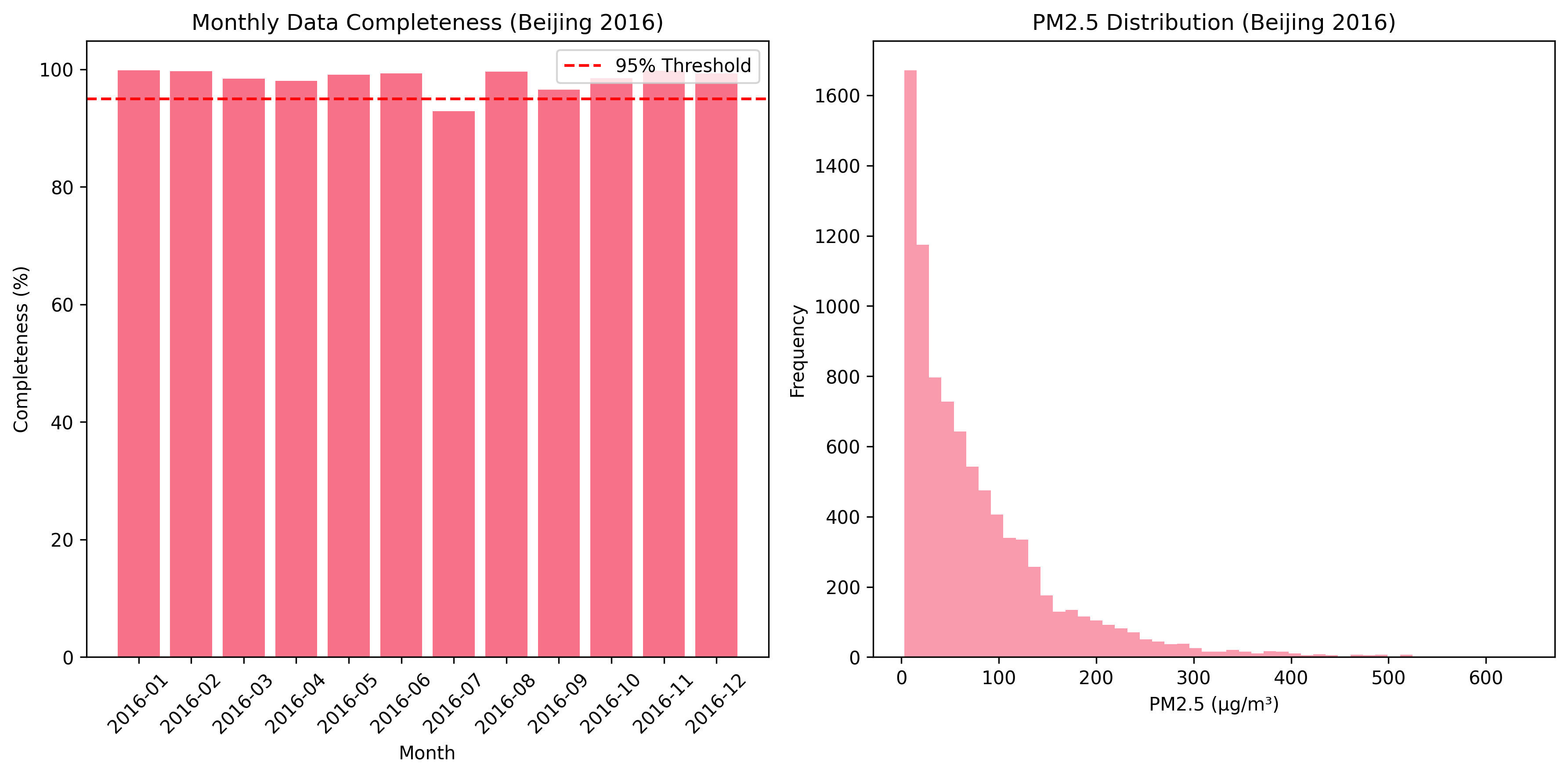


**Figure S4. Beijing PM2.5 data characteristics:** (left panel) Monthly completeness percentages, (right panel) Concentration distribution. Data from Guanyuan monitoring station (2016).


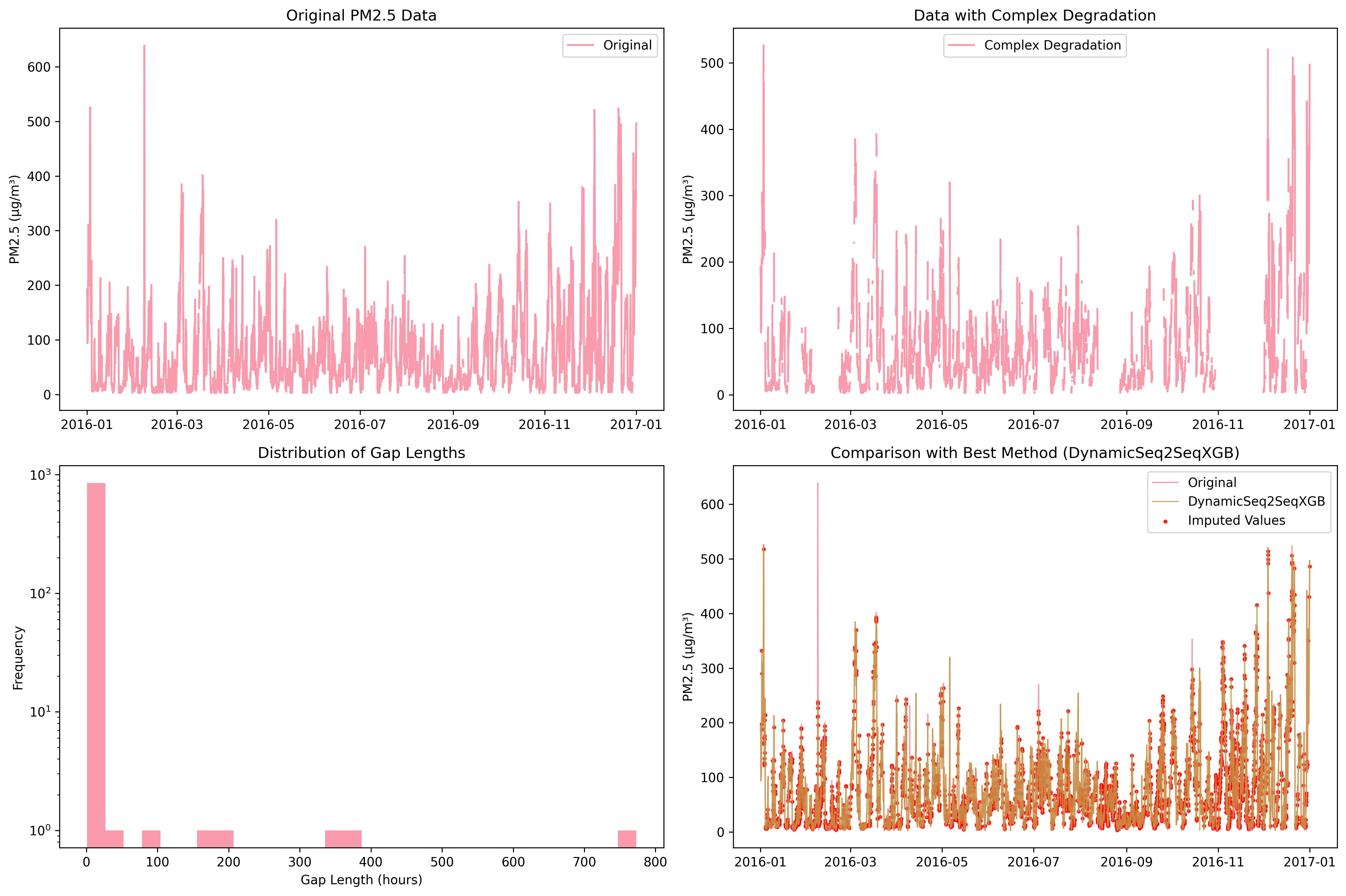


**Figure S5.** Complex degradation analysis: (top left) Original PM2.5 time series, (top right) Artificially degraded series (50% completeness), (bottom left) Synthetic gap length distribution, (bottom right) DynamicSeq2SeqXGB imputation performance.


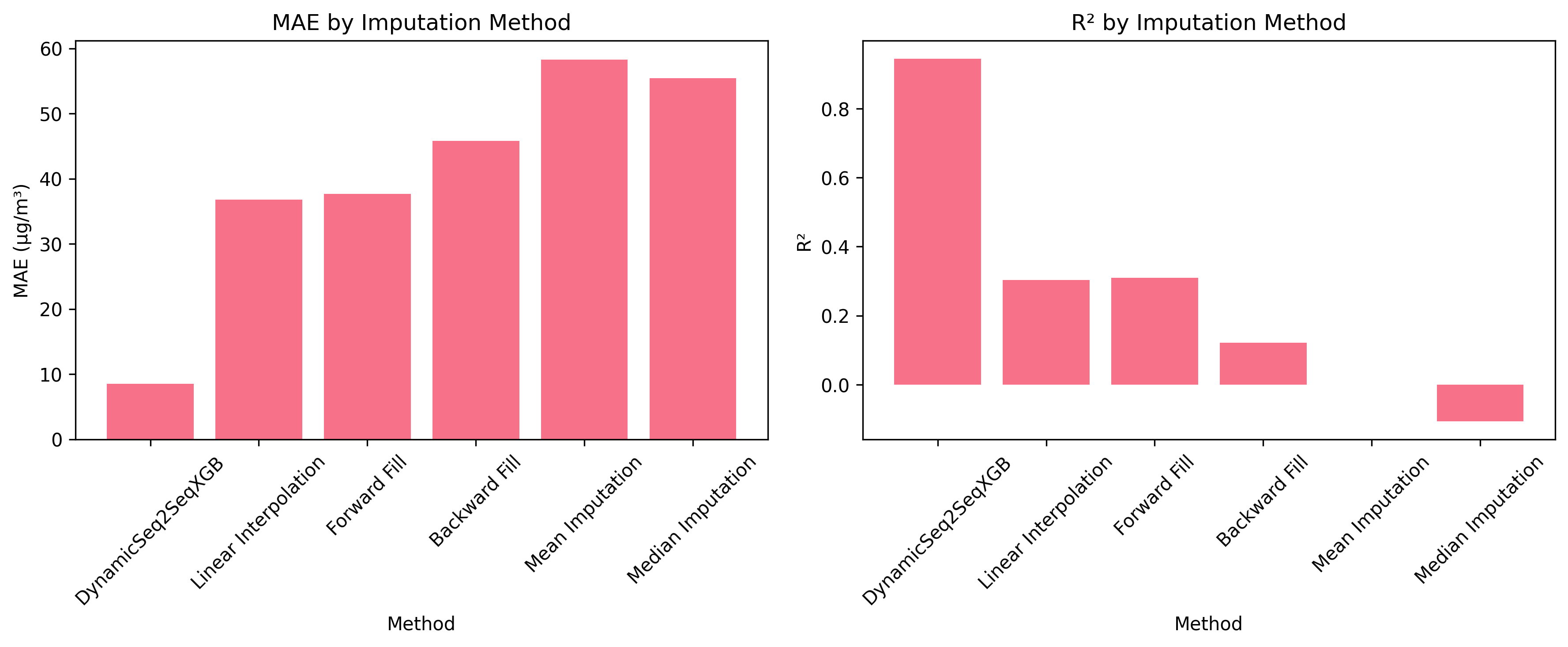


**Figure S6.** **Quantitative benchmarking of imputation methods:** (left panel) Mean Absolute Error (MAE), (right panel) Coefficient of Determination (R²).


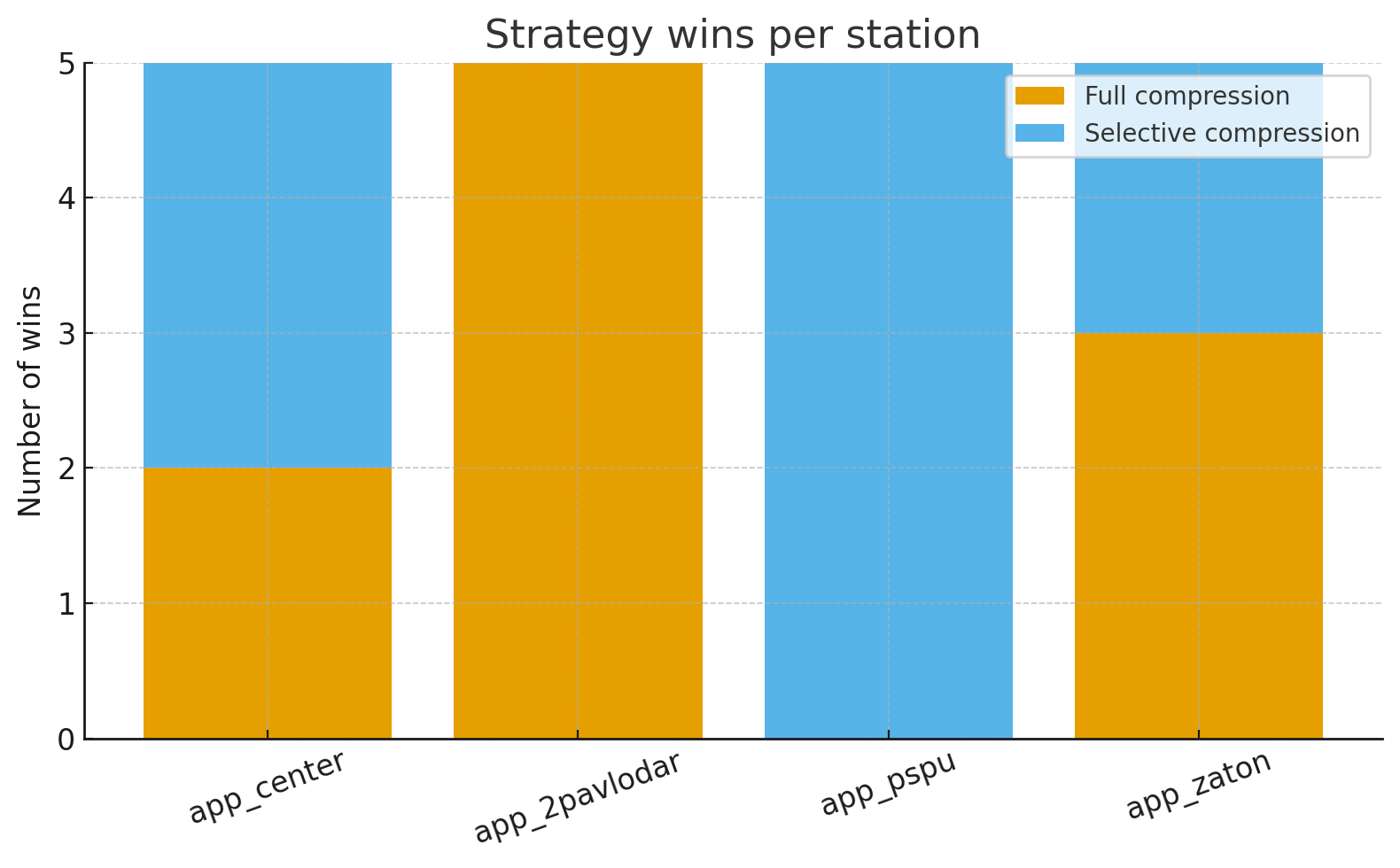


**Figure S7. Summary of station-level optimal compression strategies across five gap lengths (5, 12, 24, 48, and 72 hours).** For each station, the number of “wins” represents how many of the five gap-length scenarios yielded a lower mean absolute error (MAE) for a given compression strategy. Since lower MAE indicates better reconstruction accuracy, a higher bar segment reflects more frequent superiority of that strategy for that station. This visualization highlights persistent station-specific preferences, with some stations consistently favoring full compression while others consistently benefit from selective compression.
