# Supplementary material for: DynamicSeq2SeqXGB for PM2.5 imputation in extremely sparse environmental monitoring networks
Source: PLoS One. 2025 Dec 11;20(12):e0338788. doi: 10.1371/journal.pone.0338788 (PMC12697988; doi:10.1371/journal.pone.0338788)
Supplement: S2 File — Technical Protocol 1: Step-by-step description of the outlier detection and statistical filtering pipeline for air quality data. Technical Protocol 2: Final hyperparameter specifications, feature engineering setup, and training configuration for the DynamicSeq2SeqXGB model. (DOCX) [file pone.0338788.s002.docx]

**Technical Protocol 1: Outlier Detection and Statistical Filtering for Air Quality Data**

***1. Outlier Detection Protocol***

**Objective**: Identify statistically anomalous PM₂.₅ measurements.

**Step 1: Interquartile Range (IQR) Filter**

- Calculate quartiles:

$$\text{Q1}={25}^{\text{th}} \text{percentile},\quad\text{Q3}={75}^{\text{th}} \text{percentile}$$

- Compute IQR:

$$\text{IQR}=\text{Q3}-\text{Q1}$$

- Define bounds:

$$\text{Lower Bound}=\text{Q1}-1.5\times\text{IQR},\quad\text{Upper Bound}=\text{Q3}+1.5\times\text{IQR}$$

- Flag values outside bounds as outliers.

**Step 2: Z-Score Validation**

- Calculate station-specific statistics:

$$\mu=\text{mean},\quad\sigma=\text{standard deviation}$$

- Compute Z-score for each observation:

$$z=\frac{x-\mu}{\sigma}$$

- Flag values where (|z| > 3) as outliers.

**Step 3: Consensus Outlier Labeling**

- Mark points identified by **either IQR or Z-score** as outliers.
- Replace outliers with `NaN` for downstream processing.

***2. Statistical Filtering Protocol***

**Objective**: Ensure temporal continuity and data completeness.

**Step 1: Monthly Completeness Threshold**

- For each station and month:

$$\text{Completeness (\%)}=\left( 1-\frac{\text{\#}\text{ Missing}\text{/}\text{Outlier Hours}}{\text{Total Hours}} \right)\times100$$

- Discard months with completeness < **70%**.

**Step 2: Consecutive Valid Hours Requirement**

- Within retained months, identify segments of consecutive valid hours (non-`NaN`).
- Require at least one segment of **≥24 consecutive valid hours**.
- Discard months failing this criterion.

**Step 3: Final Dataset Construction**

- Aggregate retained months across all stations.
- Output: Curated dataset for modeling (`df_hourly_filtered.xlsx`).

***3. Validation Metrics***

- **Completeness Improvement**:

$$\Delta_{\text{comp}}=\text{Post-Filtering Completeness}-\text{Original Completeness}$$

- **Data Retention**:

$$\text{Retention (\%)}=\frac{\text{\#}\text{ Retained Hours}}{\text{Total Original Hours}}\times100$$

**Technical Protocol 2: Final Hyperparameter Specifications and Feature Engineering Setup for the DynamicSeq2SeqXGB Model**

**1. Model Architecture Overview**

**Algorithm**: Dynamic Sequence-to-Sequence XGBoost (DynamicSeq2SeqXGB)

**Base Estimator**: XGBRegressor wrapped in MultiOutputRegressor

**Model Type**: Multivariate time series imputation with dynamic context sizing

**2. Core Hyperparameters**

**2.1 XGBoost Base Configuration**

XGBRegressor(

n_estimators=50,           # Number of boosting rounds

random_state=42            # Reproducibility seed

# Other parameters use XGBoost default values

)

**2.2 Multi-Output Configuration**

MultiOutputRegressor(

estimator=XGBRegressor(...),

n_jobs=None # Single-threaded processing

)

**2.3 Beijing Dataset Configuration**

For external validation on Beijing Guanyuan dataset:

XGBRegressor(

n_estimators=100,          # Increased for external validation

max_depth=6,               # Maximum tree depth

learning_rate=0.1,         # Step size shrinkage

random_state=42            # Reproducibility seed

)

**3. Context Window Specifications**

**3.1 Fixed Context Parameters**

- **Pre-context length**: `32 hours` (context before gap)
- **Post-context length**: `32 hours` (context after gap)
- **Maximum context size**: `C_max = 32` (padding reference)

**3.2 Dynamic Context Sizing Logic**

C_dynamic = min(gap_length * 3 if gap_length <= 10 else 32, pre_context_length)

**Effective context sizes by gap length**:

- Gap 5h: `C_dynamic = min(15, 32) = 15 hours`
- Gap 12h: `C_dynamic = min(36, 32) = 32 hours`
- Gap 24h: `C_dynamic = min(32, 32) = 32 hours`
- Gap 48h: `C_dynamic = min(32, 32) = 32 hours`
- Gap 72h: `C_dynamic = min(32, 32) = 32 hours`

**4. Feature Engineering Setup**

**4.1 Input Features**

**Primary target**: `pm25_hourly` (PM₂.₅ concentrations, μg/m³)

**Exogenous features Pavlodar stations** (6 variables):

- `Ff`: Wind speed (m/s)
- `DD`: Wind direction (degrees)
- `air_temperature`: Ambient temperature (°C)
- `air_humidity`: Relative humidity (%)
- `hour`: Hour of day (0-23) - engineered temporal feature
- `season`: Season index (0-3) - engineered temporal feature

**Beijing station** (10 variables):

- `pm10_hourly`: PM₁₀ concentrations (μg/m³)
- `so2_hourly`: SO₂ concentrations (μg/m³)
- `no2_hourly`: NO₂ concentrations (μg/m³)
- `co_hourly`: CO concentrations (μg/m³)
- `o3_hourly`: O₃ concentrations (μg/m³)
- `temp_hourly`: Temperature (°C)
- `pres_hourly`: Pressure (hPa)
- `dewp_hourly`: Dew point (°C)
- `rain_hourly`: Precipitation (mm)
- `wspm_hourly`: Wind speed (m/s)
- `hour`: Hour of day (0-23)
- `day_of_week`: Day of week (0-6)
- `month`: Month (1-12)

**4.2 Temporal Feature Engineering**

# Hour extraction

df['hour'] = df['date'].dt.hour

# Season encoding (meteorological seasons)

df['season'] = (df['date'].dt.month % 12 // 3).astype(int)

# 0: Dec-Feb (Winter), 1: Mar-May (Spring)

# 2: Jun-Aug (Summer), 3: Sep-Nov (Autumn)

**4.3 Data Preprocessing Pipeline**

- **Temporal Reindexing**: Create complete hourly time series with `pd.date_range`
- **Missing Value Removal**: Drop rows with NaN in any feature for training
- **Standardization**: Apply `StandardScaler()` to all features
- **Feature Indexing**: Use indices `[0, 1, 2, 3, 4, 5, 6]` for scaled feature array

**5. Data Splitting Protocol**

**5.1 Time-Based Split**

def time_based_split_3(X_left, X_right, y, train_size=0.8):

split_idx = int(n_samples * 0.8)

# First 80% for training, last 20% for testing

**Split configuration**:

- **Training set**: First 80% of temporal sequence
- **Test set**: Last 20% of temporal sequence
- **No shuffling**: Preserves temporal order

**6. Gap Length Configuration**

**6.1 Target Gap Lengths**

gap_lengths = [5, 12, 24, 48, 72] # hours

**6.2 Synthetic Gap Generation**

- **Missing fraction**: `0.05` (5% of data converted to gaps)
- **Gap placement**: Random selection in dense data regions
- **Minimum valid sequence**: `pre_context_length + gap_length + post_context_length`
- **Reproducibility**: `random_state` parameter controls gap placement

**7. Input Tensor Construction**

**7.1 Feature Tensor Shape**

For each training sample:

input_dimensions = (

C_max * n_features + # Left context (flattened): 32 * 7 = 224

C_max * n_features + # Right context (flattened): 32 * 7 = 224

3 # Metadata: [gap_length, C_dynamic, position_mod]

)

# Total input size: 224 + 224 + 3 = 451 features

**7.2 Output Tensor Shape**

output_dimensions = max(gap_lengths) # 72(padded with zeros for shorter gaps)

**7.3 Padding Strategy**

- **Context padding**: Zero-padding to standardize context window sizes
- **Output padding**: Zero-padding to standardize output sequence lengths
- **Constant values**: `constant_values=0` for all padding operations

**8. Training Configuration**

**8.1 Cross-Gap Training**

- **Multi-gap learning**: Single model trained on all gap lengths simultaneously
- **Data combination**: Vertically stack samples from different gap lengths
- **Output masking**: Only first `gap_length` positions used for each sample

**8.2 Evaluation Protocol**

- **Number of runs**: `n_runs = 10` (statistical robustness)
- **Random seed sequence**: `random_state = [0, 1, 2, ..., 9]`
- **Metric aggregation**: Mean across all runs for final performance

**9. Outlier Detection and Filtering**

**9.1 Statistical Outlier Removal**

- **IQR filter**: Remove values outside `[Q1 - 1.5*IQR, Q3 + 1.5*IQR]`
- **Z-score filter**: Remove values with `|z-score| > 3`
- **Consensus approach**: Flag outliers identified by either method

**9.2 Temporal Filtering**

- **Monthly completeness threshold**: ≥70%
- **Consecutive validity requirement**: ≥24 consecutive valid hours per month
- **Final dataset**: `df_hourly_filtered.xlsx` (selective) or `df_hourly_merged.xlsx` (full)

**10. Model Persistence and Deployment**

**10.1 Model Artifacts**

# Model saving

joblib.dump(model, "station_name_corrected_multiseq2seq_xgb_model.joblib")

joblib.dump(scaler, "station_name_scaler.joblib")

**10.2 Feature Requirements for Inference**

- **Input columns**: `['pm25_hourly', 'Ff', 'DD', 'air_temperature', 'air_humidity', 'hour', 'season']`
- **Scaling**: Must apply same `StandardScaler` fitted during training
- **Context requirements**: Minimum 32 hours before and after gap for optimal performance

**11. Performance Validation**

**11.1 Evaluation Metrics**

- **MAE**: Mean Absolute Error (primary metric)
- **RMSE**: Root Mean Square Error
- **R²**: Coefficient of determination
- **MAPE**: Mean Absolute Percentage Error

**11.2 Benchmarking Protocol**

- **Synthetic gap testing**: Create artificial gaps in dense data sequences
- **Cross-station validation**: Test on all 5 monitoring stations
- **Gap length sensitivity**: Evaluate performance across all target gap lengths
- **Statistical significance**: Report results with confidence intervals from 10 independent runs
