# Supplementary material for: DynamicSeq2SeqXGB for PM2.5 imputation in extremely sparse environmental monitoring networks
Source: PLoS One. 2025 Dec 11;20(12):e0338788. doi: 10.1371/journal.pone.0338788 (PMC12697988; doi:10.1371/journal.pone.0338788)
Supplement: Attachment — Submitted filename: REVIEW.docx [file pone.0338788.s003.docx]

**REVIEW**

**of the article:**

**"DYNAMICSEQ2SEQXGB FOR PM2.5 IMPUTATION IN EXTREMELY SPARSE ENVIRONMENTAL MONITORING NETWORKS"**

The article is devoted to improving methods for restoring data on the concentration of pollutants in atmospheric air based on measurements taken at monitoring stations in Kazakhstan. The topic is relevant because atmospheric air pollution in populated areas increases health risks for the population, causing a number of chronic diseases. This creates a need for reliable information on atmospheric air pollution levels, both in real time and retrospectively. The accuracy and completeness of information about the current quality of atmospheric air determines the development of management decisions to prevent risks to public health.

Unfortunately, even modern and reliable devices for measuring dust and gas concentrations cannot guarantee continuous operation due to a number of independent circumstances, which inevitably leads to gaps in the source data. Depending on the nature of the missing data, this can significantly affect the calculation of average concentrations, as well as the comparative analysis of data from different monitoring stations and the calculation of comprehensive air pollution indices. At the same time, today, both in the EU countries and in the post-Soviet countries, including Kazakhstan, there are no regulatory documents, standards, or methodological recommendations regarding the procedure for restoring lost data, since this is a rather complex and non-trivial procedure. These are the tasks addressed by the authors of this publication.

This publication contains a significant number of literary sources for analyzing modern and effective approaches to processing preliminary data and methods for restoring gaps. The work considers both traditional approaches to solving the problem of missing data based on the use of statistical interpolation methods (linear interpolation, moving average, direct or filling, etc.) and more progressive and specific methods based on machine learning. Emphasis is placed on the problem of extremely sparse input data, where the use of traditional recovery methods may have low accuracy or be completely inappropriate. In this regard, the authors of the work propose using machine learning methods based on the improvement and combination of Seq2Seq and XGBoost models to validate and extend the data imputation method.

The input data for the research consists of time series of PM2.5 concentrations in the atmospheric air from five monitoring stations in Pavlodar. The database contains more than 50,000 hourly observations over 15 months, which, from a statistical point of view, is a sufficiently relevant sample for testing the proposed imputation methods. At the same time, the completeness of the data was low, ranging from 23.4% to 57.5%. In the process of preliminary statistical processing, filtering (<995 ppm) and data clipping (selective and complete) were used. After preliminary processing, classical data filling methods based on linear interpolation, moving average, forward and backward filling, as well as machine learning based on the combined Dynamic Seq2Seq XGBoost model were used. Four statistical indicators were used to evaluate the effectiveness of the model: MAE, RSME, MAPE, and the coefficient of determination, which allowed us to select the most optimal data imputation model depending on the nature of the gaps.

The analysis showed that the Dynamic Seq2Seq XGBoost model was quite effective compared to classic gap filling methods. The results of the analysis are presented in the form of tables, graphs, and diagrams, the input data array is available, and the author's DynamicSeq2SeqXGBoost model is available on the git-hub repository.

Conclusion. After reviewing this work, we were left with a very positive impression. This publication is a complete scientific work that addresses the pressing issue of correctly filling in lost monitoring data. The main content of the article is presented in a scientific style, with material presented in an easy-to-understand manner, relevant graphics, and accurate references to the sources used. The work contains all the necessary components of a scientific publication. The work is research-oriented and scientifically novel, consisting of methodological approaches to the data recovery procedure based on an improved model. The practical application of the research results concerns the implementation of the approach proposed by the authors in monitoring atmospheric air, including other priority pollutants (carbon monoxide, sulfur dioxide, nitrogen oxides, ozone, etc.), which will contribute to raising public awareness of the dangers of atmospheric air pollution.

Therefore, I believe that this work can be recommended for publication in the scientific journal PLOS One.

Buchavyi Yurii, PhD (biology)

Associate Professor,

The Department of Ecology and

Technologies of Environmental Protection,

Dnipro University of Technology
